# Enhanced expression of cell-surface B-cell receptor-associated protein 31 contributes to poor survival of non-small cell lung carcinoma cells

**DOI:** 10.1371/journal.pone.0188075

**Published:** 2017-11-16

**Authors:** Se-Ri Seo, Hyun Min Lee, Hong Seo Choi, Won-Tae Kim, Eun-Wie Cho, Chun Jeih Ryu

**Affiliations:** 1 Department of Integrative Bioscience and Biotechnology, Institute of Antiancer Medicine Development, Sejong University, Gwangjin-gu, Seoul, Korea; 2 Epigenomics Research Center, Korea Research Institute of Bioscience and Biotechnology, Yuseong-gu, Daejeon, Republic of Korea; University of South Alabama Mitchell Cancer Institute, UNITED STATES

## Abstract

B-cell receptor-associated protein 31 (BAP31) is an endoplasmic reticulum (ER) membrane protein which plays a role as a molecular chaperone for the newly synthesized transmembrane proteins. BAP31 is also an important apoptosis regulator for extrinsic apoptosis induction in the ER membrane. Recent studies have shown that BAP31 is also expressed on the surface of embryonic stem cells. However, the function of cell surface BAP31 (csBAP31) still remains unclarified. In an attempt to search for surface markers on tumorspheres, here, we generated monoclonal antibodies (MAbs) against the sphere cells from the non-small cell lung carcinoma cell (NSCLC) line A549. SP1-B7, one of the MAbs, recognized csBAP31 whose expression was further increased on A549 sphere cells, as compared with A549 adherent cells. To investigate the role of csBAP31 in A549 cells, A549 adherent and sphere cells were stained with annexin V, propidium iodide, and SP1-B7. Interestingly, annexin V-high cells showed increased expression of csBAP31 as compared with annexin V-low cells. Caspase-3/7 activity was also increased in csBAP31-high cells as compared with csBAP31-low cells, suggesting that csBAP31-high cells are more sensitive to apoptosis. To further demonstrate the survival of csBAP31-positive A549 cells, csBAP31-positive and -negative A549 cells were sorted and subjected to the clonogenic survival assay. The colony number of csBAP31-positive A549 cells was decreased by approximately 1.7-fold, as compared that of csBAP31-negative A549 cells, suggesting that csBAP31-positve cells are sensitive to cell death indeed. The results suggest that enhanced expression of csBAP31 contributes to poor survival of NSCLC cells.

## Introduction

B-cell receptor-associated protein 31 (BAP31) is a 28 kDa endoplasmic reticulum (ER) membrane protein, and regulates the fate of various ER membrane proteins as a molecular chaperone [1–4]. In addition to its original role as an ER chaperone and quality control factor, BAP31 also plays a critical role in apoptosis induction. BAP31 interacts with procaspase-8L and Bcl-2/Bcl-xL on the ER membrane [4, 5]. BAP31 is cleaved by caspase-8, and its cleaved product, p20, is an important inducer of apoptosis [4, 5]. Although BAP31 is mainly localized to the ER membrane, recent studies have shown that BAP31 is also present on the cell surface of human embryonic stem cells (hESCs) [6–8]. It seems that cell surface BAP31 (csBAP31) promotes cell survival through the regulation of cell adhesion to extracellular matrix in hESCs [6, 7, 9]. However, the function of csBAP31 on cancer cells still remains unclarified.

Sphere culture was originally used to isolate neural stem cells and expanded to enrich and characterize various adult stem cells and cancer stem cells (CSCs) [10–12]. The enrichment of CSCs through tumorsphere cultivation is very simple and does not require a background knowledge on cell surface markers. In this study, we employed the sphere culture system to enrich CSCs from the non-small cell lung carcinoma (NSCLC) cell line A549. In an attempt to search novel surface markers on CSCs, we used A549 adherent cells as decoy immunogen and generated monoclonal antibodies (MAbs) that showed increased binding activity to cell surface antigens on A549 sphere cells by the decoy immunization strategy [13]. SP1-B7, one of the MAbs, bound to some NSCLC cell lines but not to peripheral blood mononuclear cells (PBMC). SP1-B7 also showed increased binding activity to the sphere cells from the hepatocellular carcinoma cell line Huh7 cells. SP1-B7 antigen turned out to be BAP31 by mass spectrometry and Western blot analysis. Subsequent studies revealed that enhanced expression of csBAP31 contributes to poor survival of NSCLC cells, contrary to our initial expectation. The results show for the first time the function of csBAP31 on cancer cells and suggest csBAP31 as a putative pro-apoptotic flag on cancer cells.

## Materials and methods

### Cell culture

Human NSCLC cell lines (A549, NCI-H460 and NCI-H1703) were purchased from the Korean Cell Line Bank (KCLB, Seoul, Korea) and maintained in RPMI-1640 medium supplemented with 10% fetal bovine serum (FBS) and antibiotic-antimycotic solution (Life Technologies, Seoul, Korea). Human hepatocellular carcinoma cell line Huh7 was also purchased from the KCLB. Human bronchial epithelial cell line BEAS-2B was purchased from the American Type Culture Collection (ATCC, Manassas, VA) and maintained in DMEM medium supplemented with 10% FBS and antibiotic-antimycotic solution (Life Technologies). H9 hESCs were cultured on mouse embryonic fibroblast (MEF) feeder cells as described previously [6, 14]. Human PBMCs were isolated by the Ficoll-Paque Plus method (GE Healthcare, Seoul, Korea). FO myeloma cells were maintained in DMEM medium supplemented with 10% FBS and antibiotic-antimycotic solution (Life Technologies)

### Tumorsphere culture

To generate tumorspheres, adherent cells were dissociated by Trypsin-EDTA (Welgene, Daegu, Korea) and filtered through 40 μm strainer and centrifuged at 1500 rpm for 3 min at room temperature (RT). Cells were seeded at 2.1 x 10^3^ cell/cm^2^ in ultra-low attachment plate (Corning, Seoul, Korea) and maintained in DMEM/F12 (Corning) supplemented with 20 ng/ml fibroblast growth factor 2 (R&D system, Seoul, Korea), 20 ng/ml epidermal growth factor (PeproTech, Seoul, Korea) and 1 x B27 supplement (Life technologies). The medium was replaced every 3 days. Sphere cells were dissociated by enzyme-free cell dissociation solution (Millipore, Seoul, Korea) when surface expressed proteins were analyzed by flow cytometry.

### Generation and purification of MAbs

Murine MAbs were generated as previously described [15]. Briefly, 1 x 10^6^ cells of A549 sphere cells were injected into the hind footpads of 5 female Balb/c mice (DBL, Chungbuk, Korea). To generate a panel of hybridomas producing MAbs that bind to A549 sphere cells but not to adherent A549 cells, adherent A549 cells were immunized into the right hind footpads of mice as decoy immunogen, while A549 sphere cells were immunized into the left hind footpads of the same mice 3 days later as target immunogen. The lymphocyte suspension from the left popliteal lymph nodes was fused to FO myeloma cells. Isotype analysis of selected antibodies was carried out by Mouse Immunoglobulin Isotyping Kit (BD Biosciences, Seoul, Korea), according to the supplier’s protocol. MAbs were purified from the culture supernatants of hybridomas by Protein G-Sepharose column chromatography as described before [15, 16].

### Flow cytometry

Flow cytometric analysis was performed as described previously [14]. Briefly, cells were treated with dissociation solution (Millipore) and suspended in PBA (1% bovine serum albumin, 0.02% NaN_3_ in phosphate buffered saline (PBS), pH 7.4). Cells were then incubated with primary antibodies for 30 min at 4°C and further incubated with fluorescein isothiocyanate (FITC)-conjugated anti-mouse IgG (Santa Cruz Biotechnologies, Santa Cruz, USA) or FITC-conjugated anti-rabbit IgG (Santa Cruz Biotechnologies). After washing with PBA, propidium iodide (PI)-negative cells were analyzed for the antibody binding using FACSCalibur (BD Biosciences). For the analysis of annexin V-positive and csBAP31-positive A549 cells, A549 cells were first stained with SP1-B7 and phycoerythrin (PE)-conjugated anti-mouse IgG (Life technologies). Cells were further stained with annexin V-FITC (BD Biosciences) and PI before analysis. The paired t-test was a statistical procedure used to compare the difference between the mean fluorescence intensities (MFI) of two populations, and a *p*-value of less than 0.05 was considered statistically significant.

### Cell-surface biotinylation, immunoprecipitation, and Western blotting

Cell surface biotinylation, immunoprecipitation, and Western blotting were performed as described previously [6, 16]. Briefly, biotin-labeled A549 adherent and sphere cells were treated with lysis buffer (25 mM Tris-HCl, pH 7.5, 250 mM NaCl, 5 mM EDTA, 1% Nonidet P-40, 2 μg/ml aprotinin, 100 μg/ml PMSF, 5 μg/ml leupeptin, 1mM NaF, and 1mM NaVO_3_) at 4°C for 30 min, and nuclei were removed by centrifugation. To immunoprecipitate the antigens recognized by the antibodies, the precleared lysates with agarose beads were incubated with isotype control antibody, a commercially available rabbit polyclonal anti-BAP31 antibody (α-BAP31), or SP1-B7 at 4°C overnight, and further incubated with Protein G agarose beads at 4°C for 3 h. The bound proteins were eluted from the beads by heating at 100°C for 10 min or by acidic solution (0.1M Glycine-HCl, pH 2.8), and neutralized by alkaline solution (1.0M Tris-HCl, pH 9.0) after extensive washing with lysis buffer. Eluted proteins were fractionated by SDS-PAGE and then transferred to a nitrocellulose membrane for Western blotting. The membrane was blocked in 5% skim milk in Tris-buffered saline with 0.1% Tween-20 at RT for 1 h, and was incubated with streptavidin-horseradish peroxidase (SA-HRP, GE Healthcare, Seoul, Korea) at RT for 1 h. The immunoblots were visualized with Western blot detection system (iNtRON Biotechnology, Seongnam, Korea). For Western blot analysis, the proteins on polyacrylamide gels were transferred to a nitrocellulose membrane and incubated with SP1-B7 or α-BAP31 followed by HRP-conjugated anti-mouse IgG or anti-rabbit IgG for 1h at RT. Glyceraldehyde 3-phosphate dehydrogenase (GAPDH) was used as a loading control. The immunoblots were visualized as described above.

### Mass spectrometry

The protein of interest was enzymatically digested in-gel in a manner similar to that described previously [17]. The search program, ProFound, developed by The Rockefeller University (http://129.85.19.192/profound_bin/WebProFound.exe), was used for protein identification by peptide mass fingerprinting [18]. Spectra were calibrated with trypsin auto-digestion ion peak *m/z* (842.510, 2211.1046) as internal standards.

### Caspase-3/7 activity assay

CellEvent Caspase 3/7 Green Flow Cytometry Assay Kit (Thermo Fisher Scientific) was used to measure caspase activity in A549 cells according to the manufacturer’s instructions. Briefly, after incubation of A549 cells with or without 1 mM H_2_O_2_ for 5 min at 37°C, A549 cells were washed and incubated with SP1-B7 and PE-conjugated anti-mouse IgG (Thermo Fischer Scientific) at RT for 10 min in PBA. The A549 cells were then incubated with 500 nM CellEvent Caspase 3/7 Green Detection Reagent and 1 μM SYTOX AADvanced Dead Cell Stain (Thermo Fisher Scientific) for 30 min at 37°C. The A549 cells were analyzed using FACSCalibur (BD Biosciences). The paired t-test was a statistical procedure used to compare the difference between the MFIs of two populations, and a *p*-value of less than 0.05 was considered statistically significant.

### Cell sorting

A549 cells were dissociated by dissociation solution (Millipore) and filtered through 40 μm strainer and centrifuged at 1500 rpm for 3 min. Thirty-four million cells were incubated with 70 μg of SP1-B7 for 30 min at RT and washed by PBS (pH 7.4) including 5% FBS and further incubated with Alexa fluor 488-conjugated anti-mouse IgG (Life technologies) for 30 min at RT in the dark. After washes, the cells were isolated by BD FACSAria (BD Biosciences). After cell sorting, SP1-B7-positive or -negative cells were counted with 0.4% Trypan blue (Welgene) and live cells were used for the clonogenic survival assay.

### Clonogenic survival assay

After cell sorting of A549 with SP1-B7, SP1-B7-positive or -negative A549 cells were seeded at 10 cells/cm^2^ and cultured for 9 days. The medium was changed every 3 days and cells were stained with 0.5% crystal violet. Stained cells were counted by cell count program. The paired t-test was a statistical procedure used to compare the difference between two populations, and a *p*-value of less than 0.05 was considered statistically significant.

## Results and discussion

### Generation of MAb SP1-B7

**Fig 1:**
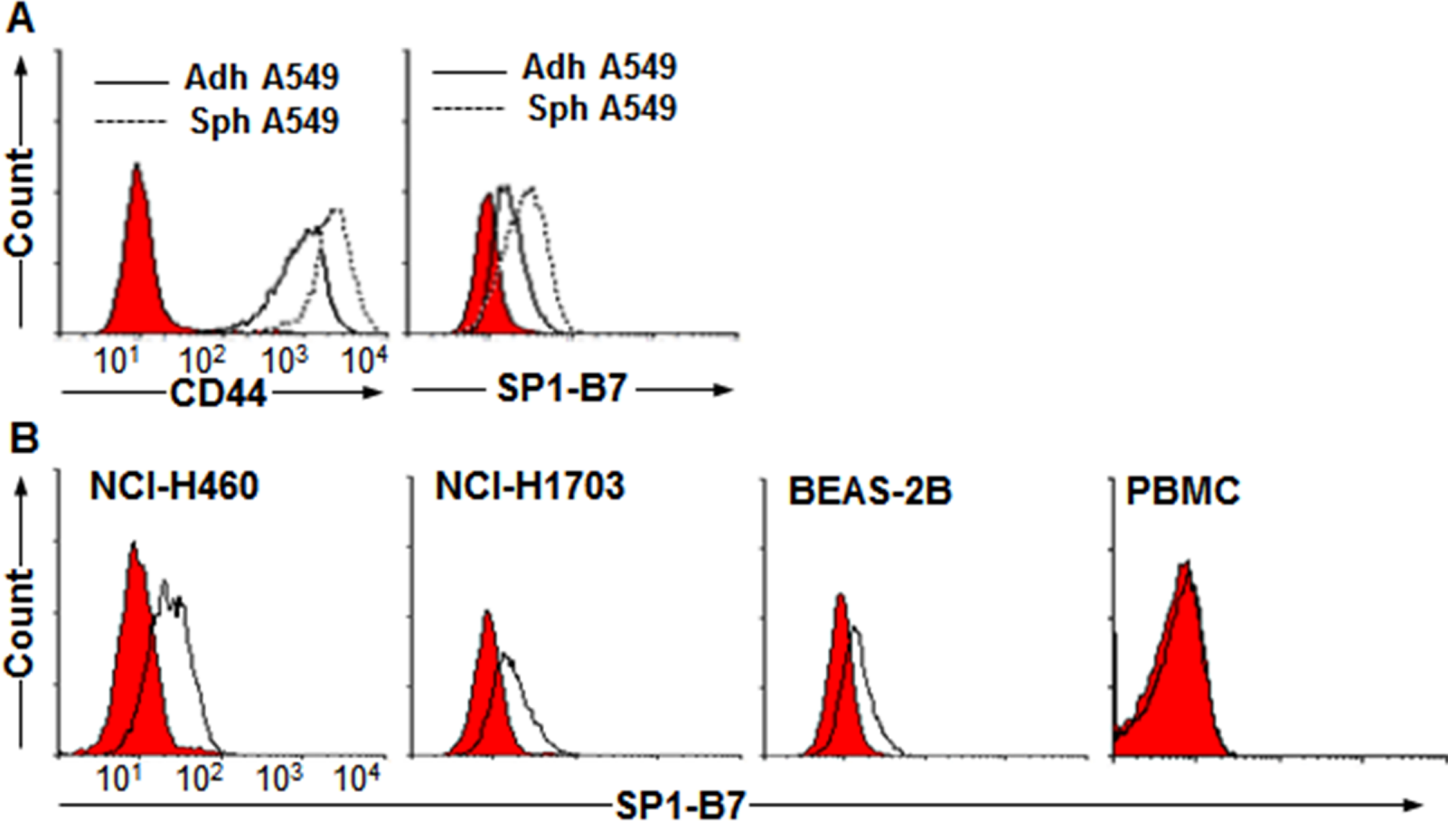
Cell surface expression of SP1-B7 antigen on various NSCLC cells. (A) Flow cytometric analysis of A549 adherent (adh A549) and sphere cells (sph A549) with SP1-B7. Red-colored population indicates secondary antibody staining as a control. (B) Flow cytometric analysis of NSCLC cell lines (NCI-H460, NCI-H1703), virus-transformed lung epithelial cell (BEAS-2B), and PBMC with SP1-B7.

Tumorsphere culture has been known as one of simple method to enrich CSCs *in vitro* [11]. Among NSCLC cell lines, A549 cells have shown high levels of cancer-initiating cells, aldehyde dehydrogenase activity and side population [19–21], suggesting that a large portion of A549 cells has CSC-like properties. Based on literatures, we postulated that CSC surface markers might be enriched on A549 sphere cells. Therefore, by using the decoy immunization strategy, we generated a panel of MAbs recognizing cell surface molecules on A549 sphere cells to apply them on searching for CSC-specific surface markers [15]. A549 sphere cells were injected into left footpads of mice as target immunogen, while adherent A549 cells were injected into right footpads of the same mice 3 days earlier as decoy immunogen (**S1 Fig**). Fusion of left popliteal lymph node cells to myeloma cells generated 6 hybridomas secreting MAbs, and two MAbs showed binding activity to the surface molecules on A549 sphere cells (**Table 1**). SP1-B7 (IgG1, κ), one of the two MAbs, bound to A549 adherent cells and showed increased binding activity to A549 sphere cells as compared to A549 adherent cells (**Fig 1A**). SP1-B7 also showed binding activity to other NSCLC cell lines NCI-H460 and NCI-H1703 (**Fig 1B**). SP1-B7 also slightly bound to the virus-transformed lung epithelial cell line BEAS-2B, but it did not bind to peripheral blood mononuclear cells (PBMCs) (**Fig 1B**). The enhanced binding activity of SP1-B7 to tumorsphere cells seems to be common because the same phenomenon was also observed in Huh7 sphere cells (**S2 Fig**).

**Table 1:** List of 6 MAbs against A549 sphere cells.

Name	Isotype	Binding reactivity by flow cytometry		
		A549	A549 Sphere	PBMC
SP1-B2	IgG, κ	-	-	-
SP1-B7	IgG, κ	+	++	-
SP1-C4	IgG, κ	-	-	-
SP1-D2	IgG, κ	-	-	-
SP2-A5	IgG, κ	-	-	-
SP2-B4	IgM, κ	-	++	-

### SP1-B7 recognizes cell surface-expressed BAP31

**Fig 2:**
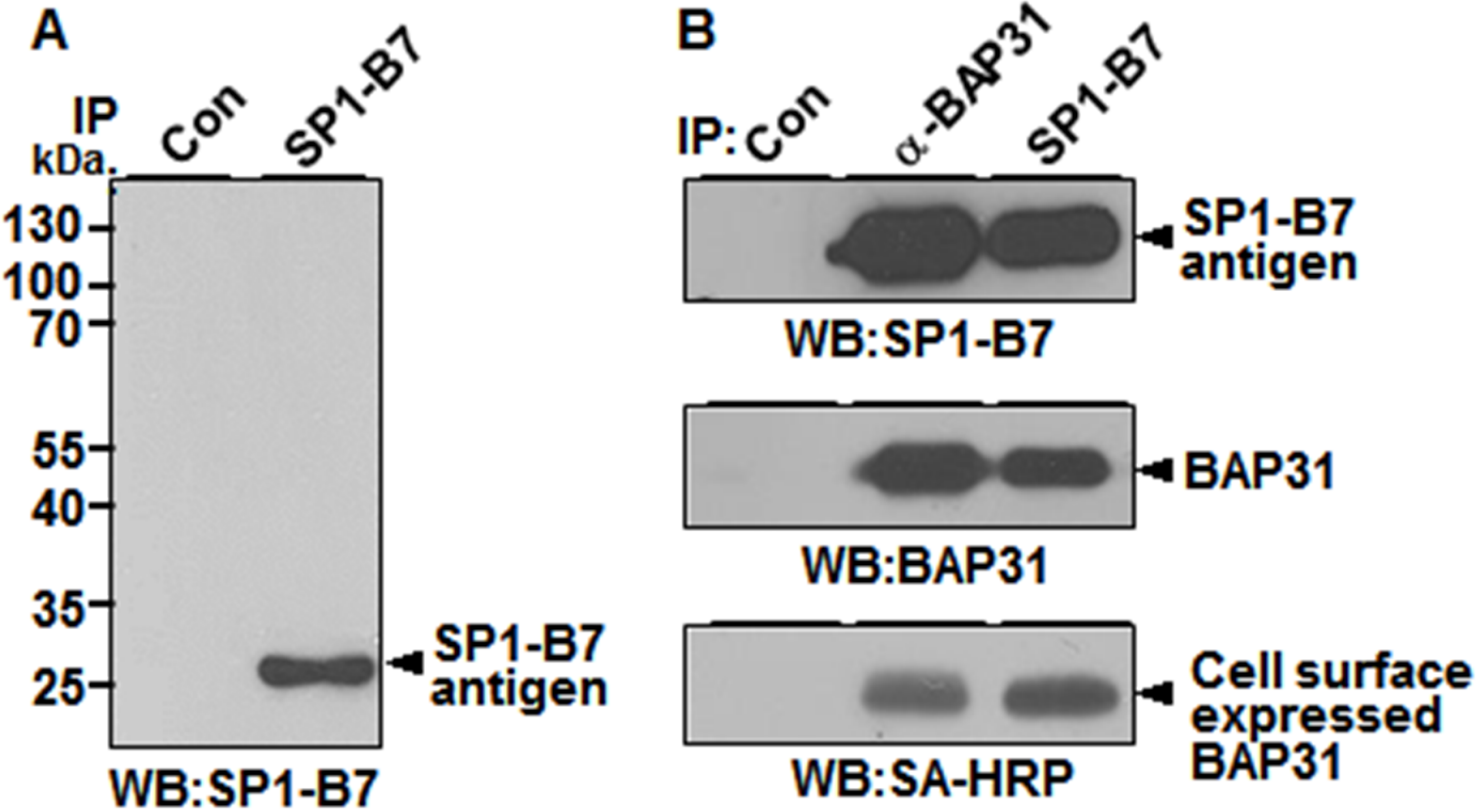
Identification of SP1-B7 antigen. (A) Immunoprecipitation of SP1-B7 antigen with SP1-B7. Cell lysates from A549 adherent cells were immunoprecipitated with isotype control antibody (Con) or SP1-B7, and the immunoprecipitates were Western blotted with SP1-B7. (B) Immunoprecipitation of biotinylated A549 cell lysates with isotype control antibody, α-BAP31, and SP1-B7. Cell surface proteins of A549 adherent cells were biotinylated and subjected to immunoprecipitation. Immunoprecipitates were detected by Western blots with the indicated antibodies or SA-HRP.

To identify the cell surface antigen recognized by SP1-B7, A549 cell lysates were immunoprecipitated with SP1-B7, and the immnuprecipitates were analyzed by Western Blot analysis with SP1-B7. SP1-B7 recognized an approximately 28 kDa protein in A549 cells (**Fig 2A**). The 28 kDa protein was excised and subjected to mass spectrometry. Mass spectrometric analysis of the 28 kDa protein identified it as BAP31 from a protein database search (**S3 Fig**). To confirm that BAP31 is expressed on the cell surface of A549 cells, the cell surface molecules of A549 cells were biotinylated and subjected to immunoprecipitation and Western blotting with SP1-B7 and α-BAP31, a commercially available rabbit polyclonal antibody against BAP31. Proteins immunoprecipitated with α-BAP31 or SP1-B7 were readily detected by Western blot analysis with SP1-B7 or α-BAP31 (**Fig 2B**, the first and second panels), indicating that SP1-B7 recognizes the BAP31 protein indeed. The immunoprecipitated BAP31 proteins were also detected by SA-HRP (**Fig 2B**, the third panel), indicating that the BAP31 proteins recognized by the antibodies are expressed on the cell surface. Thus, the results indicate that SP1-B7 recognizes cell surface-expressed BAP31.

### csBAP31 is upregulated on tumorspheres

**Fig 3:**
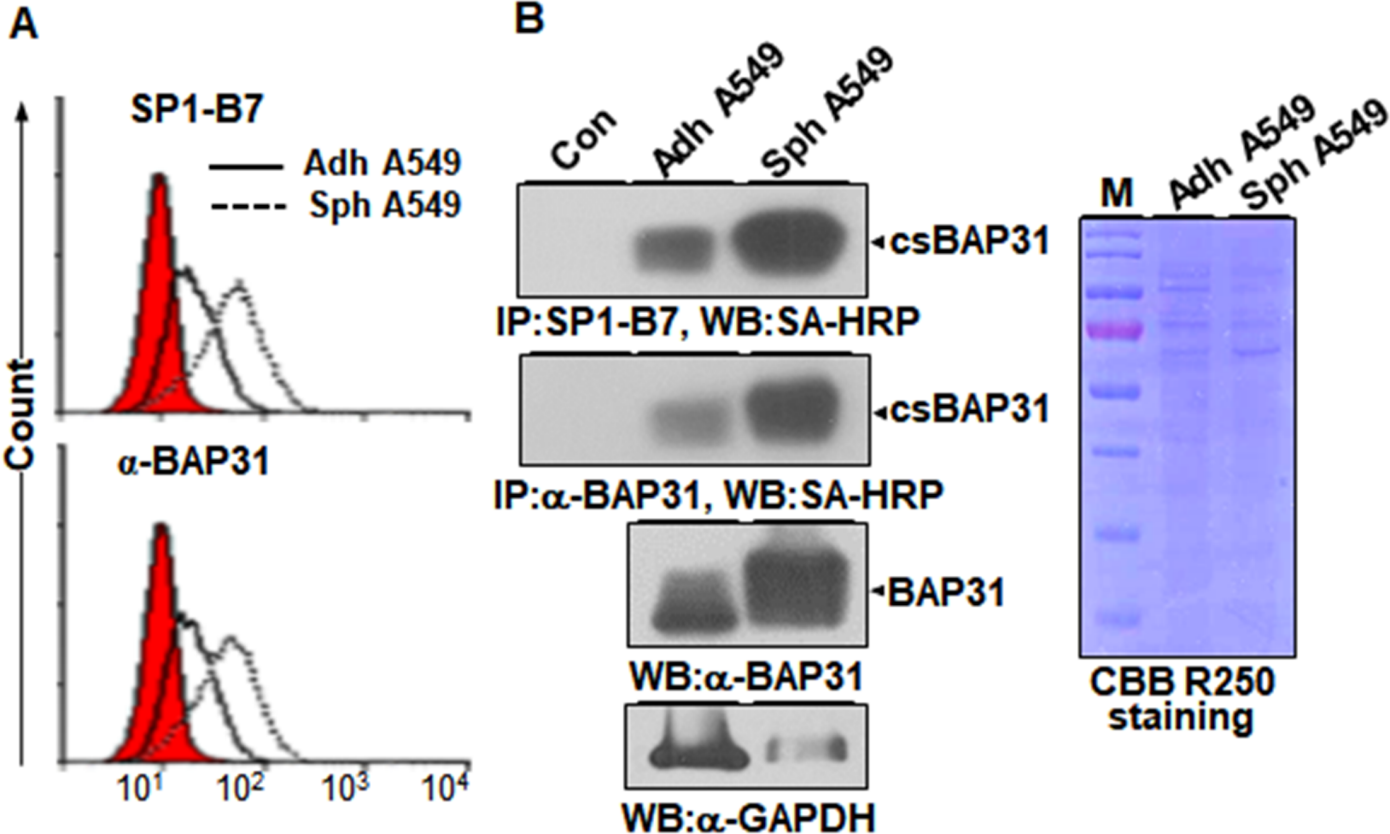
csBAP31 is upregulated on tumorspheres. (A) Flow cytometric analysis of A549 adherent (adh A549) and sphere cells (sph A549) with SP1-B7 and α-BAP31. Red-colored population indicates secondary antibody staining as a control. (B) Immunoprecipitation of csBAP31 with SP1-B7 and α-BAP31. Cell surface proteins of A549 adherent and sphere cells were biotinylated and subjected to immunoprecipitation with isotype control antibody (Con), SP1-B7, or α-BAP31, and immunoprecipitated csBAP31 proteins were detected with SA-HRP (top and middle panels). Total BAP31 proteins were also detected in two cell lysates with α-BAP31 (lower panel). GAPDH was used as a loading control. Coomassie brilliant blue (CBB) staining of two cell lysates is also shown as a loading control (right panel).

To compare the expression levels of csBAP31 between A549 adherent and sphere cells, A549 adherent and sphere cells were analyzed by flow cytometry with SP1-B7 and α-BAP31. SP1-B7-reactive csBAP31 was upregulated in A549 sphere cells (**Fig 3A**, upper panel). The similar result was also obtained with α-BAP31, a commercially available anti-BAP31 antibody, suggesting that csBAP31 is upregulated on A549 sphere cells, as compared to A549 adherent cells (**Fig 3A**, lower panel). To further compare the expression levels of csBAP31 between A549 adherent and sphere cells, cell surface proteins of the same number of A549 adherent and sphere cells were biotinylated, subjected to immunoprecipitation with SP1-B7 and α-BAP31, and detected with SA-HRP. Increased expression of csBAP31 was detected with SP1-B7 and α-BAP31 in A549 sphere cells (**Fig 3B**, left top and middle panels). Total BAP31 protein was also increased in A549 sphere cells (**Fig 3B**, left lower panel), suggesting that the increased expression of csBAP31 is due to increased expression of total BAP31 protein. Thus, the results indicate that csBAP31 is upregulated on A549 sphere cells, as compared with A549 adherent cells.

### csBAP31 is upregulated on annexin V-high and caspase 3/7-high A549 cells

**Fig 4:**
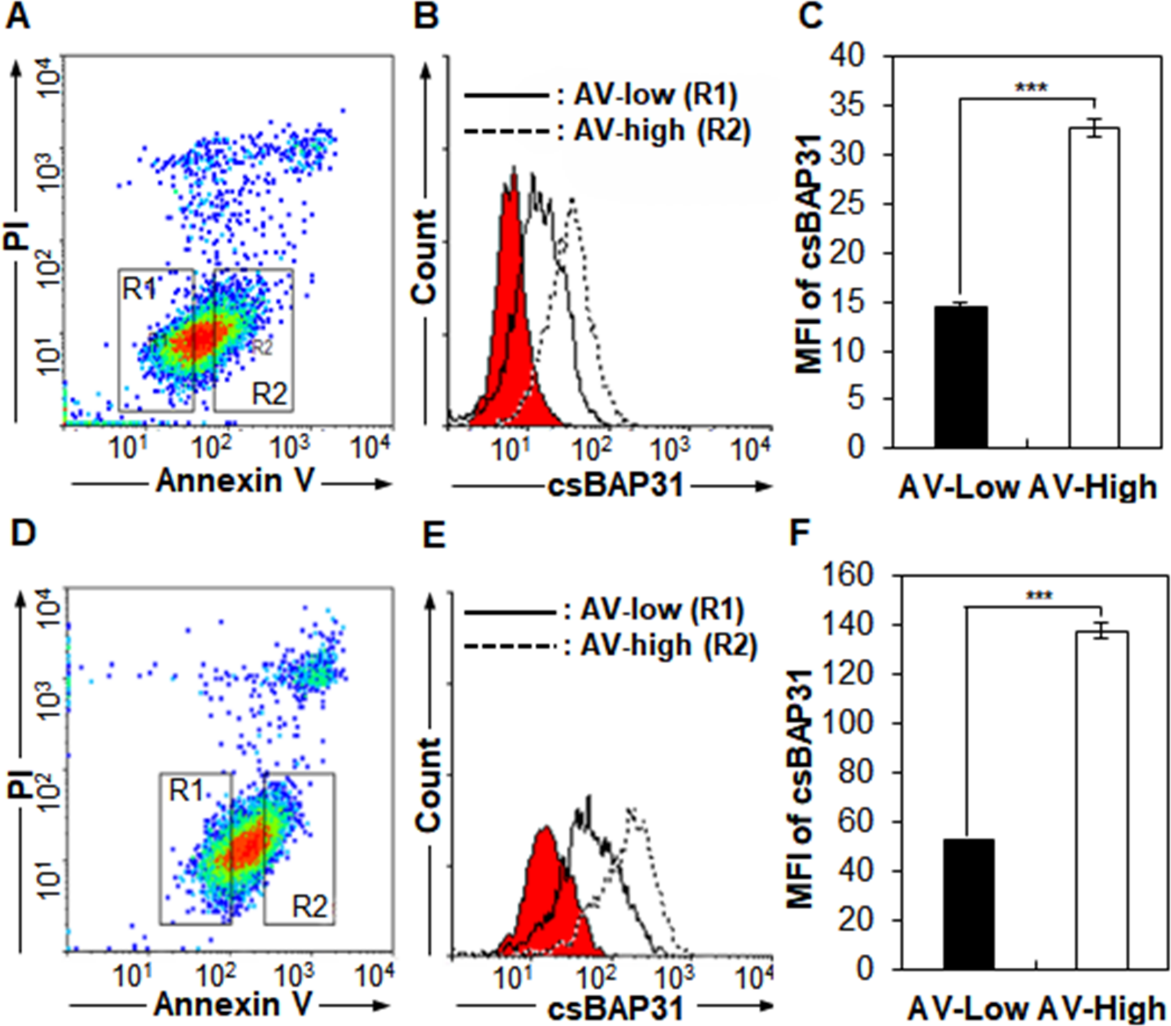
csBAP31 is upregulated on annexin V-high A549 cells. A549 adherent (A, B, C) or sphere cells (D, E, F) were stained with SP1-B7, annexin V, and PI. To examine whether csBAP1 expression is associated with apoptosis, annexin V-low (R1) and -high cells (R2) were gated on PI-negative cells (A, D) and analyzed for the expression of csBAP31 (B, E). csBAP31 expression is presented as MFIs (*n = 3*) (C, F). ***, *p*<0.005.

Studies have shown that BAP31 is a target of extrinsic apoptosis induction and regulates cell survival in hESCs and mESCs [5, 6, 9, 22]. Therefore, csBAP31 may be also involved in cell survival on A549 cells. To investigate the role of csBAP31 on A549 cells, A549 adherent cells were detached and stained simultaneously with annexin V, PI, and SP1-B7. Interestingly, csBAP31 was upregulated in annexin V-high A549 cells as compared to annexin V-low A549 cells (**Fig 4A, 4B and 4C**). The similar results were obtained with A549 sphere cells (**Fig 4D, 4E and 4F**). The same phenomenon was also obtained with hESCs where csBAP31 are expressed on the cell surface [6–8] (**S4 Fig**). Thus, the results suggest that enhanced cell surface expression of csBAP31 may contribute to apoptosis.

**Fig 5:**
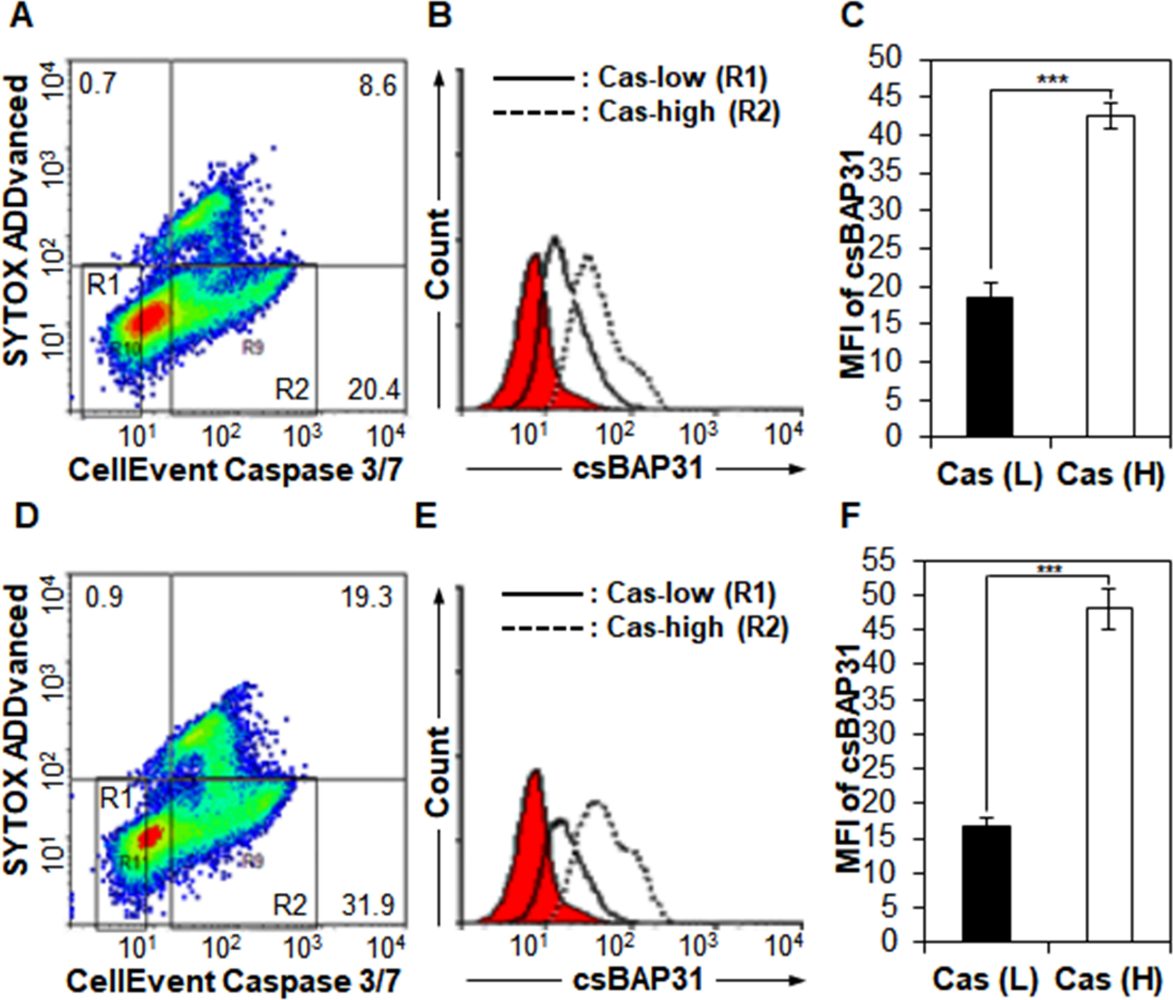
csBAP31 is upregulated on caspase 3/7-high A549 cells. After incubation of A549 cells without (A, B, C) or with H_2_O_2_ (D, E, F), A549 adherent cells were detached and incubated with SP1-B7 and PE-conjugated anti-mouse IgG. The A549 cells were then incubated with caspase 3/7 flow cytometry assay kit before analysis. To examine whether csBAP31 expression is associated with early apoptosis, caspase-low (R1) and -high cells (R2) were gated on SYTOX AADvanced-negative cells (A, D) and analyzed for the expression of csBAP31 (B, E). csBAP31 expression is presented as MFI (*n = 3*) (C, F). ***, *p*<0.005.

To further prove whether csBAP31 is really upregulated in the early stages of apoptotic cells, A549 adherent cells were incubated with or without H_2_O_2_ to induce early apoptosis before detachment. After incubation of A549 cells with SP1-B7, the levels of spontaneous and induced apoptosis were measured in csBAP31-low and -high A549 cells by using caspase 3/7 flow cytometry assay kit. csBAP31 expression was increased by approximately 2.3-fold in caspase 3/7-high A549 cells (Cas-high) as compared to caspase 3/7-low A549 cells (Cas-low) (**Fig 5A, 5B and 5C**). When A549 cells were treated with H_2_O_2,_ csBAP31 expression was further increased by approximately 2.9-fold in caspase 3/7-high A549 cells (**Fig 5D, 5E and 5F**). The results further prove that higher cell surface expression of BAP31 is positively associated with increased apoptosis.

### csBAP31-positive cells show decreased survival

**Fig 6:**
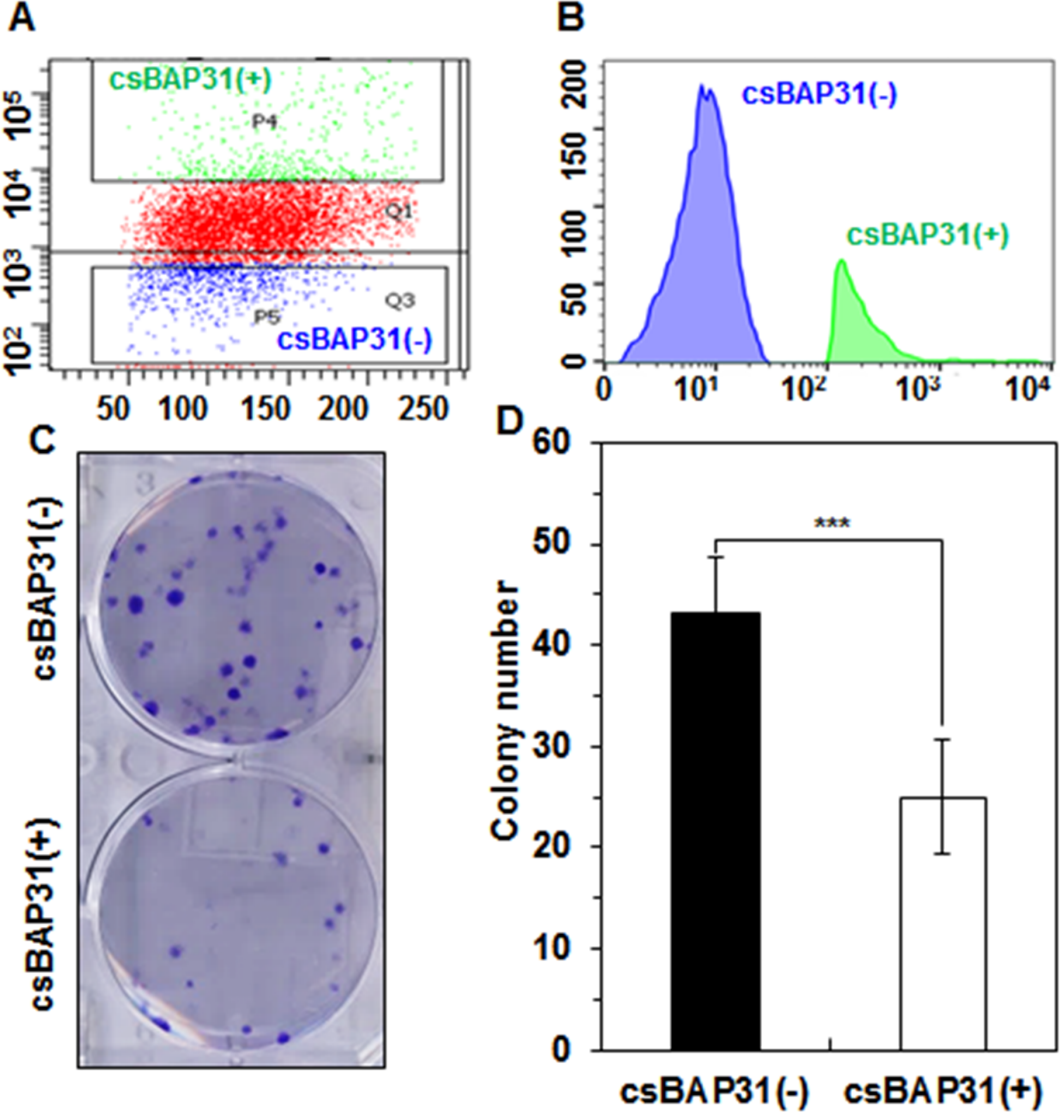
csBAP31-positive cells show decreased survival. (A, B) csBAP31-positive and -negative A549 cells were sorted after staining with SP1-B7. (C) Live sorted cells were seeded and cultured for 9 days. Colonies were stained with crystal violet. (D) Statistical analysis of C (*n* = 10). ***, *p*<0.005.

Although annexin V staining represents the early stages of apoptosis, the early apoptotic process is reversible [23]. To further dissect the role of csBAP31 in A549 cells, csBAP31-positive and -negative A549 cells were sorted and subjected to the clonogenic survival assay (**Fig 6A and 6B**), because the clonogenic survival assay is a method to determine the survival and growth of cells in cancer cell lines under the stress of low density seeding. csBAP31-positive A549 cells showed reduced clonogenic survival by approximately 1.7-fold, as compared with csBAP31-negative A549 cells (**Fig 6C and 6D**). The result suggests that enhanced cell surface expression of csBAP31 contributes to decreased survival in NSCLC cells indeed.

By using MAb SP1-B7 recognizing BAP31, we accidently found that BAP31 is expressed on the surface of NSCLC cells, and is further increased on NSCLC sphere cells. The observation seems to be common in cancers cells because the same results were also obtained with Huh7 sphere cells. In the very beginning, we thought that csBAP31 was induced under serum starvation and detachment stresses during tumorsphere culture, and enhanced expression of csBAP31 might have anti-apoptotic and pro-proliferative activity on cancer cells. The reason why we thought so was due to the fact that BAP31 knockdown causes increased apoptosis and decreased proliferation in hESCs [6]. Contrary to our initial speculation, however, csBAP31 was upregulated in annexin V- and caspase-high A549 cells, and csBAP31-high A549 cells showed decreased cell survival in the clonogenic survival assays (**Figs 4, 5 and 6**). The phenomenon seems to be common because csBAP31 was also upregulated in annexin V-high hESCs (**S4 Fig**). Thus, the present study shows that enhanced cell surface expression of BAP31 contributes to decreased cell survival, in contrast to the previous study in which BAP31 expression was positively associated with cell survival in hESCs [6]. However, the earlier study didn’t show clear distinction between cytoplasmic and cell surface BAP31 expression. To make sure the role of csBAP31 in A549 cells, in this study, we clearly distinguished csBAP31-positive A549 cells from csBAP31-negative A549 cells by cell sorting (**Fig 6A and 6B**). The results suggest that csBAP31-positive A549 cells are more prone to cell death, and suggest csBAP31 as a putative pro-apoptotic flag on cancer cells.

This finding is reminiscent of the role of cell-surface GRP78 in cancer cells because GRP78 plays a role as an ER chaperone and are exposed on the cell surface. It appears that cell-surface GRP78 plays a role as a receptor for pro-survival or apoptotic signaling in cancer cells [24, 25]. Pharmacological induction of cell surface GRP78 induces the process of apoptosis, and is used as a therapeutic modality for cancer treatment [26, 27]. Although GRP78 translocation from the ER to the cell surface is not clearly understood, it is believed that various stresses promote GRP78 relocation on the cell surface [24, 28]. Similar to cell surface GRP78, the results of the present study also suggest that csBAP31 induction is an indicator of pro-apoptosis in NSCLC cells because csBAP31-positive cells are more sensitive to cell death. How BAP31 translocation from the ER to the cell surface is not clear yet, but the present study suggests that serum starvation and detachment stress may promote BAP31 relocation on the cell surface, because csBAP31 was upregulated in the tumorsphere cells. How BAP31 is translocated to cell surface and take part in cell death in NSCLC cells is the interesting next subject to study.

## Supporting information

S1 Fig Morphologies of A549 adherent and sphere cells. The scale bar is 200 κm.
(TIF)

S2 Fig Flow cytometric analysis of Huh7 adherent (adh Huh7) and sphere cells (sph Huh7) with anti-EpCAM, anti-ALDH1A1, and SP1-B7. (TIF)

S3 Fig Mass spectrometric identification of SP1-B7 antigen after immunoprecipitation with SP1-B7. The 28-kDa bands from A549 cell lysates were treated with trypsin, and the resulting peptides were analyzed by peptide mass fingerprinting. Six tryptic peptides (underlined) originating from the 28-kDa protein matched BAP31.
(TIF)

S4 Fig csBAP31 is upregulated on annexin V-high hESCs. (A, B) H9 hESCs were stained with SP1-B7, annexin V, and PI. To examine whether csBAP1 expression is associated with apoptosis, annexin V-low (R1) and -high cells (R2) were gated on PI-negative cells and analyzed for the expression of csBAP31.
(TIF)
