# Supplementary figures and images for: Enhanced expression of cell-surface B-cell receptor-associated protein 31 contributes to poor survival of non-small cell lung carcinoma cells
Source: PLoS One. 2017 Nov 16;12(11):e0188075. doi: 10.1371/journal.pone.0188075 (PMC5695096; doi:10.1371/journal.pone.0188075)

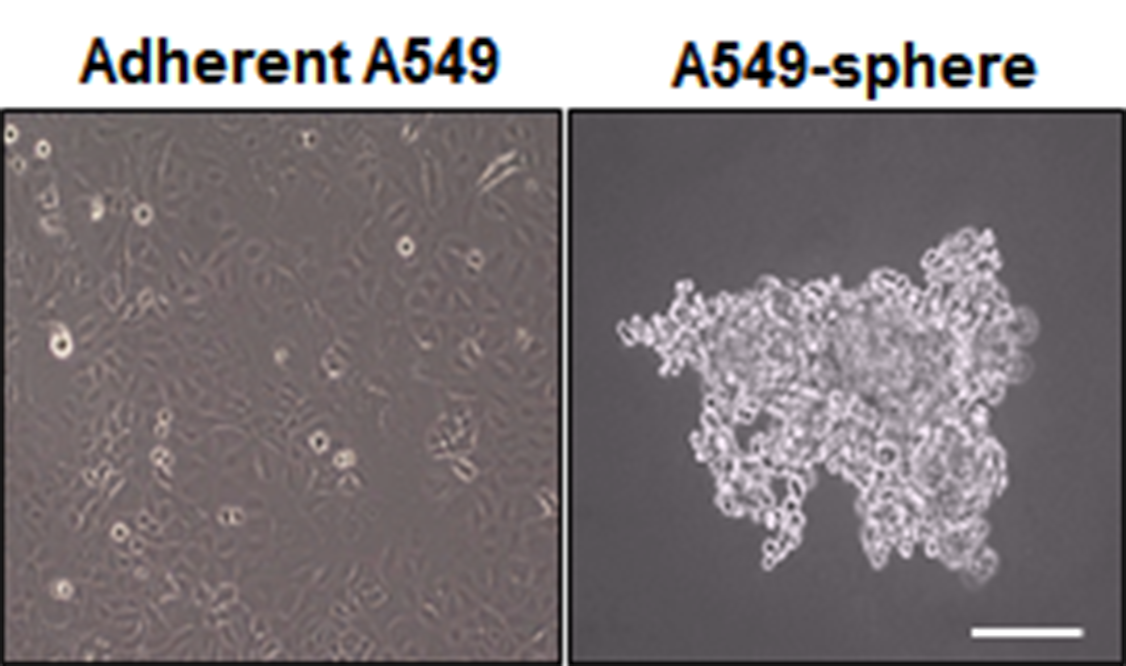

Supplement: S1 Fig — The scale bar is 200 κm. (TIF) [file pone.0188075.s001.tif]

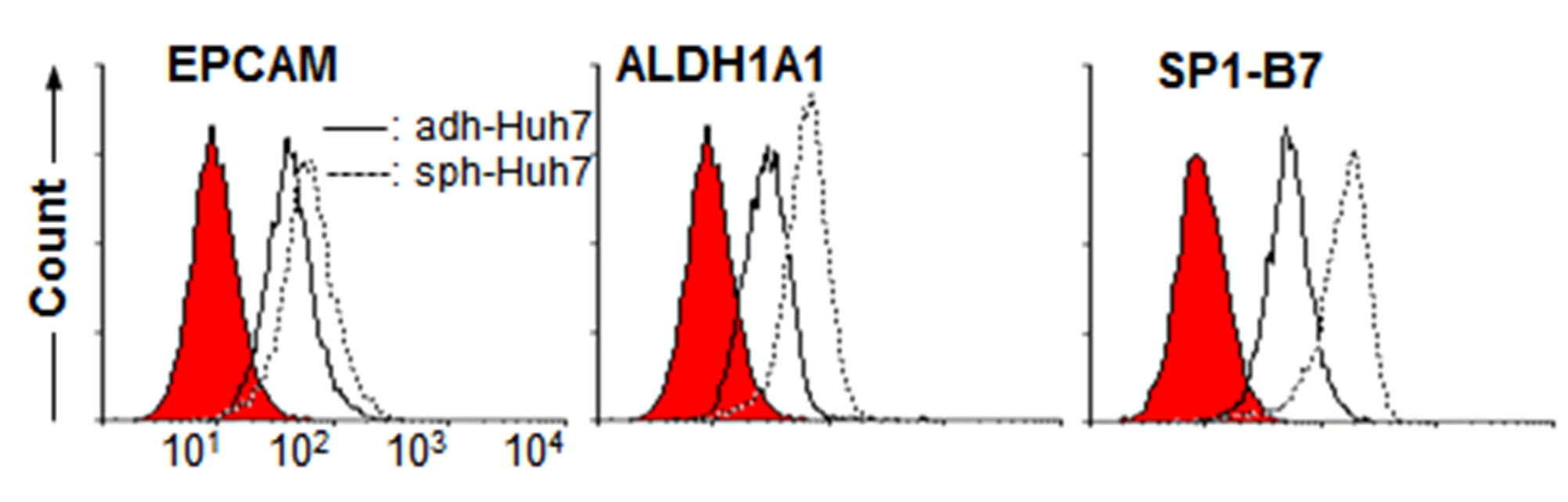

Supplement: S2 Fig — (TIF) [file pone.0188075.s002.tif]

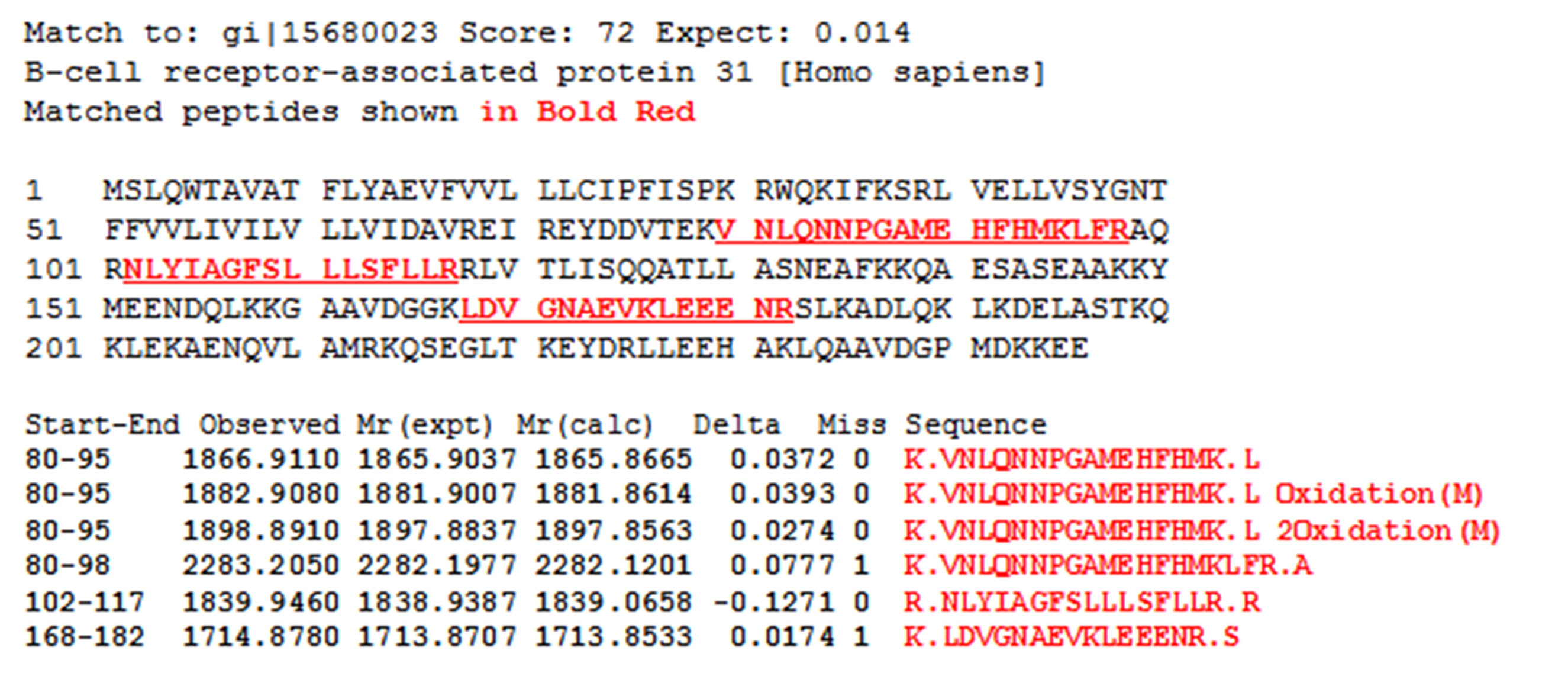

Supplement: S3 Fig — The 28-kDa bands from A549 cell lysates were treated with trypsin, and the resulting peptides were analyzed by peptide mass fingerprinting. Six tryptic peptides (underlined) originating from the 28-kDa protein matched BAP31. (TIF) [file pone.0188075.s003.tif]

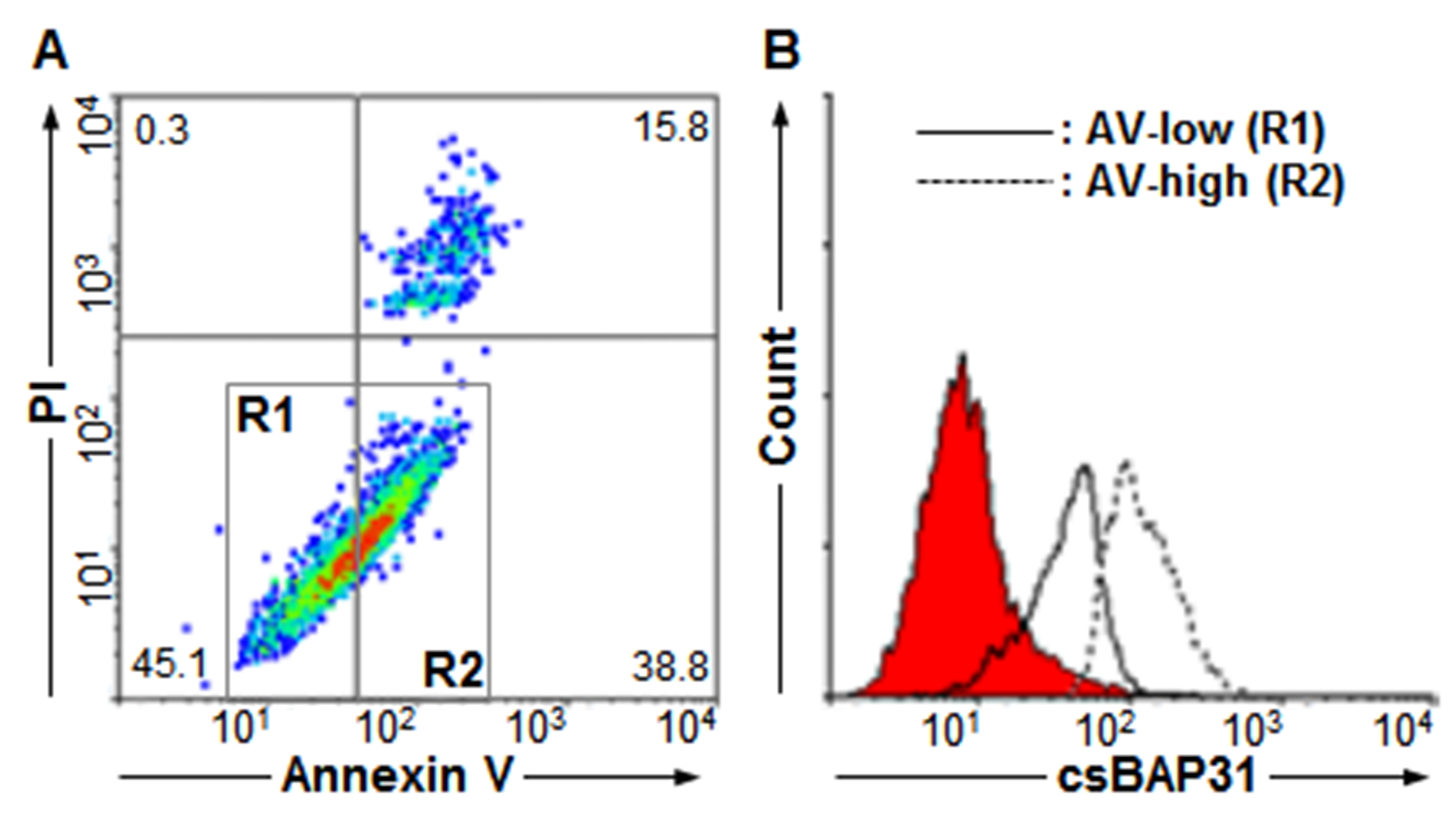

Supplement: S4 Fig — (A, B) H9 hESCs were stained with SP1-B7, annexin V, and PI. To examine whether csBAP1 expression is associated with apoptosis, annexin V-low (R1) and -high cells (R2) were gated on PI-negative cells and analyzed for the expression of csBAP31. (TIF) [file pone.0188075.s004.tif]
